# Chinese Mothers’ Intent to Disclose Their HIV Status to Their Children: The Role of Positive Outcome Expectations and Reward Responsiveness

**DOI:** 10.3389/ijph.2023.1605559

**Published:** 2023-05-09

**Authors:** Qian Wang, Kam Hei Hui, Ailing Wang, Xiaoyan Wang, He Sun, Stephanie Tsz Yung Lau, Changhe Wang, Phoenix Kit Han Mo

**Affiliations:** ^1^ National Center for Women and Children’s Health, Chinese Center for Disease Control and Prevention, Beijing, China; ^2^ Department of Psychology, The Chinese University of Hong Kong, Hong Kong, Hong Kong SAR, China; ^3^ Centre for Health Behaviours Research, JC School of Public Health and Primary Care, The Chinese University of Hong Kong, Hong Kong, Hong Kong SAR, China

**Keywords:** HIV status disclosure, outcome expectation, reward responsiveness, behavioral activation, Chinese mothers

## Abstract

**Objective:** The current study investigated the role of positive outcome expectations and reward responsiveness in intention to disclose HIV status to children among women living with HIV in China. The moderating role of reward responsiveness was also explored.

**Method:** A 1-year longitudinal survey was conducted. 269 women living with HIV who had at least one child aged >5 years and had not yet disclosed their HIV status to their oldest child were selected from a larger sample of women living with HIV at baseline, with a total of 261 respondents completing the follow-up survey.

**Results:** After adjusting for significant socio-demographic and medical variables, positive outcome expectations positively predicted mothers’ intention to disclose HIV, while reward responsiveness had a negative effect. A moderation effect of reward responsiveness was found, with further analysis showing that reward responsiveness has strengthened the relationship between positive outcome expectations and intention to disclose HIV.

**Conclusion:** Findings support the relevance of positive outcome expectations and reward responsiveness to intention of disclosure among women living with HIV in China.

## Introduction

According to the National Center for AIDS/STD Control and Prevention of China CDC, there were 270,000 women living with HIV in China in 2020. Many women living with HIV are of reproductive age and have children. With the introduction of antiretroviral therapy (ART), many of them can live a healthy life and play a vital role in childcare. With their continued childcare role, disclosure of maternal HIV status to children has thus become a potential challenge and important public health concern [1, 2].

Self-disclosure is a process that involves self-initiated discussion of previously concealed and potentially stigmatizing information with the intention of improving one’s sense of psychological wellbeing, and/or maintaining relationships [3]. Maternal HIV disclosure to children is beneficial for both mothers and children [4]. These benefits include children’s involvement in taking care of the mother [5, 6], emotional relief, decrease in psychological distress and parenting stress, improvement in the mental health of the mother [7, 8], better planning and independence for the children [1, 6], improvement in the parent-child relationship, and reduction in child emotional and behavioral problems [8]. On the other hand, maternal HIV disclosure to children may also carry risks, such as leading children to be worried or afraid [1, 9, 10]. The World Health Organization (WHO) recommends children of school age should be informed about their parents’ HIV status, and younger children should be informed gradually, to accommodate their cognitive skills and emotional maturity [11].

Despite the numerous benefits associated with maternal HIV disclosure, low maternal HIV disclosure to children was reported [1, 2]. The prevalence of maternal HIV disclosure was only 39.0% in China, 20%–67% in the U.S., 11.6% in South Africa [12], and 11%–50% in other countries [6, 13–16]. Traditional Chinese culture does not encourage disclosure of stressful events such as maternal HIV infection [17]. Women living with HIV in China often find maternal HIV disclosure difficult due to various concerns [15, 18]. Many have a low level of confidence with regards to talking about HIV or explaining the cause of their illness to their children, and struggle with deciding when and how to disclose [15, 18].

Several theories have been developed to explain HIV disclosure behavior. For example, the Disease Progression Theory proposed that disclosure is related to HIV progression [19], and the Consequence Theory suggests that disease progression influences disclosure through perceived psychological, social, and material consequences or outcomes of informing others [19, 20]. Furthermore, the Disclosure Process Model [21, 22] specifies that a disclosure event is predicted by various inhibitors and facilitators. For example, norms against disclosure, anticipated negative reactions, motivations against disclosure, and negative/unknown serostatus of the confidant are considered inhibitors; while acceptance of HIV, time since diagnosis, guilt, hyperaccessible thoughts, social support, and importance of relationship are considered facilitators of disclosure. The model also specifies that the discloser’s perceptions of how positive or negative the disclosure event would be lead to various consequences, including psychological distress, health outcomes, and behavioral outcomes. Due to the low level of maternal HIV disclosure, prospective studies on the antecedents of maternal HIV disclosure are limited. Most studies exploring factors to maternal HIV disclosure were cross-sectional in nature [23, 24], using intention to disclose as the proxy outcome as it is believed that intention would be the most proximal determinant of behavioral performance [25, 26].

Outcome expectations are perceived physical, social, and other consequences that will result from performing a behavior [27]. They strongly influence behaviors [28] and are a central construct of a number of theories for HIV disclosure, including the Consequence Theory [20, 29], Social Cognitive Theory [28], and Disclosure Process Model [21, 22]. While positive outcome expectations would result in a higher intention, negative outcome expectations would result in a lower intention to act. It has been shown that a belief that disclosure of HIV will improve family relationships is a typical positive outcome expectation, while perceived rejection from the child and negative impact on the parent-child relationship and child outcomes are typical negative outcome expectations for parental HIV disclosure [30]. Outcome expectations were found to be associated with HIV disclosure to sex partners among HIV-positive MSM [20, 28, 29, 31–35]. One study among women living with HIV in China has shown that negative outcome expectations were negatively associated with, while positive outcome expectations were positively associated with, intention to disclose indirectly through disclosure self-efficacy [23]. Furthermore, positive outcome expectations had a stronger association with disclosure intention than negative outcome expectations, suggesting that increasing perceptions of positive outcomes of disclosure might be more impactful [23].

The extant literature has highlighted the importance of studying the interaction between belief (cognition) and motivation emotion/physiological reaction in driving health behavior [36, 37]. Reward responsiveness, defined as one’s ability to experience pleasure in the anticipation and presence of reward-related stimuli, can be one important motivational factor that moderates the effect of outcome expectation. Reward responsiveness is subsumed in the behavioral activation system, which corresponds to one’s motivation to approach goal-oriented stimuli. According to the regulatory focus theory, there are two types of regulatory focus: promotion and prevention [38, 39]. People with a promotion focused motivational system are more concerned with accomplishment, occurrence of reward and the presence of positive consequences. It was found to predict adaptive functioning, psychological wellbeing, emotion regulation, and health behaviors such as physical activity [40–42]. People with higher reward responsiveness respond more positively to the presence of a reward. Therefore, it is conjectured that reward responsiveness may strengthen the effect of positive outcome expectations on disclosure intention among women living with HIV.

### The Present Study

Using a 1-year longitudinal study, the present study examined the relationship between outcome expectations and HIV status disclosure intention among women living with HIV, and the moderating role of reward responsiveness on the relationship between outcome expectations and intention to disclose HIV. It is hypothesized that positive outcome expectations would predict HIV status disclosure intention. Furthermore, reward responsiveness would moderate the association between positive outcome expectations and disclosure intention, such that the association between outcome expectations and disclosure intention would be stronger among those with higher reward responsiveness.

## Methods

### Participants

The present study utilized data from a sub-sample of women living with HIV selected from a larger sample of women living with HIV. Participants for the larger sample were recruited from 8 antenatal care (ANC) clinics in Yunnan and Guangxi provinces in mainland China. Inclusion criteria were (i) women, (ii) 18 years and above; (iii) Chinese. These two provinces were selected as they have been the pilot areas of the implementation of mother-to-child HIV transmission prevention since 2003, and thus have a solid foundation of HIV prevention and control as well as women and children’s healthcare.

The sub-sample for the present study was selected by identifying those women living with HIV who had at least one child over 5 years old and had not yet disclosed their HIV status to her oldest child. The use of 5 years old as a cut-off was set based on the WHO’s recommendations that children of school age should be informed about their parents’ HIV status. The same cut-off has also been used in other studies of HIV disclosure [43].

### Study Procedure

The baseline study was conducted between November 2019 to January 2020. Staff of the 8 ANC clinics screened and invited eligible women to participate in the study. Those who expressed initial interest were directed to research staff in charge of the survey in the same clinic. Participants were briefed with details of the study and written informed consent was obtained from those who agreed to take part in the survey. Participants were ensured that study was voluntary and that they could withdraw from it anytime according to their will. The research staff then conducted an individual, face-to-face structured interview with the participant in a private room of the clinic. The interview took around 30 min to complete. Those who completed the baseline survey were asked to provide their contact details and were invited for a follow-up survey 1 year later at the clinic. Ethics approval for human subjects research was obtained from the authors’ institution.

Out of the 382 women living with HIV who had at least one child recruited for the larger study at baseline, 113 (29.5%) had already disclosed their HIV status to their eldest child and were excluded, the remaining 269 participants were thus included as the baseline sub-sample. A total of 261 of them completed the follow-up survey.

### Measures

#### The Following Variables Were Measured at Baseline

Socio-demographic information, including age (in years), ethnicity (Han majority or other minorities), education level (primary or below, secondary, tertiary or above), marital status (married, cohabiting, single, divorced, widowed), monthly family income (RMB 1,000, 1,001–2,000, >2,000), self-perceived health (very poor, poor, fair, good, very good), number of children, age of the eldest child (in years), and gender of the eldest child (male, female) were obtained. Medical characteristics, such as duration of HIV diagnosis (in years), mode of transmission (sexual transmission, injective drug use, unknown), and disease stage (asymptomatic, symptomatic, AIDS) were also collected.

Positive outcome expectations for HIV disclosure were measured by the positive outcome expectation subscale of the Outcome Expectation for Maternal HIV Disclosure Scale previously developed by the team [23]. The positive outcome expectations scale measures 4 dimensions: psychological relief (3 items), improved intimacy (3 items), better planning of the child (3 items), and better adjustment of the child (3 items). Participants estimated how likely each outcome would be to occur if they disclose their HIV status to their eldest child on a 5-point Scale from 1 = very unlikely to 5 = very likely. A higher score reflects higher expected likelihood for the respective outcome expectations to occur. The Cronbach’s alpha was 0.96 in the present study.

Reward responsiveness was measured by the 5-item response responsiveness subscale of the Behavioral Activation Scale (BAS). Items were rated on a 4-point Scale from 1 = very true for me to 4 = very false for me. After reverse-scoring, higher total score indicates higher capability to respond to rewards positively. The Cronbach’s alpha was 0.90 in the present study.

#### The Following Variables Were Measured at the 1-Year Follow Up

HIV status disclosure and intention to disclose were measured at 1-year follow-up. Participants were asked to indicate whether they have disclosed their HIV status to the eldest child at 1-year follow-up. Among those who have not yet disclosed, they also indicated their likelihood to disclose their HIV status to their eldest child in the future on a 5-point Scale from 1 = highly unlikely to 5 = highly likely.

### Data Analysis

In the present study, positive outcome expectations for HIV disclosure was the independent variable, reward responsiveness was the moderator, and HIV status disclosure intention was the dependent variable. A total of 4 participants disclosed their maternal HIV status during the period of study and were thus removed from the final analysis. The final sample consisted of 257 participants. Descriptive statistics (mean or percentage) of studied variables were first reported. Zero-order correlations among independent background variables and target dependent variables were obtained. The main effect of baseline positive outcome expectations and baseline reward responsiveness on follow-up HIV disclosure intention was tested with hierarchical regression analysis. Baseline socio-demographic variables and medical variables significantly related to follow-up HIV disclosure intention was entered in the first block of the hierarchical regression analysis, while baseline positive outcome expectations and baseline reward responsiveness were entered in the second block of the hierarchical regression analysis. Interaction terms between baseline positive outcome expectations and baseline reward responsiveness were added in the third block of the hierarchical regression analysis to test for the moderating effect of reward responsiveness. Missing data was handled with pairwise deletion for random mistakes in data collection. All analyses were conducted using SPSS Statistic 27.

## Results

### Background Characteristics of the Participants at Baseline

More than half of the participants (68.2%) were 40 years old or below. The majority of were from the Han ethnicity (88.8%) and were married (78.1%). One third of the participants (33.5%) received a primary education or below, and around half had a total monthly household income of RMB 2000 or below (50.1%). The majority (83.1%) of the participants have been diagnosed with HIV for more than 5 years. Most of them rated their health as fair, good or very good (95.7%) (Table 1).

**TABLE 1:** Socio-demographic characteristics of women living with HIV at baseline (N = 269) (China, 2019–2022).

	N	%
Socio-demographic variables		
Age (M, SD)	38.3	6.23
Ethnicity		
Han majority	239	88.8
Other minorities	30	11.2
Education level		
Primary or below	90	33.4
Secondary	172	64.0
Tertiary or above	7	2.6
Marital Status		
Married or cohabiting	214	79.9
Single	8	3.0
Divorced or widowed	46	17.1
Monthly household income (RMB)		
1,000	48	17.8
1,001–2,000	87	32.3
>2,000	134	49.8
Self-perceived health		
Very poor or poor	11	4.1
Fair	135	50.2
Good or very good	123	45.7
Number of children		
1	106	39.4
2	97	36.1
3	37	13.8
4 or above	29	10.8
Age of the eldest child		
5–10	100	37.2
11–20	156	58.0
>20	13	4.8
Gender of the eldest child		
Male	140	52.0
Female	129	48.0
Disease-related characteristics		
Duration of HIV diagnosis		
5 years or below	45	16.9
6–10 years	90	33.7
10 years or above	132	49.4
Self-reported mode of HIV transmission		
Sexual transmission	244	90.7
Injective drug use	12	4.5
Don’t know/do not wish to answer	13	4.8
HIV stage		
Asymptomatic	212	78.8
Symptomatic	8	3.0
AIDS	38	14.1
Uncertain	11	4.1

### Correlations Between Variables of Interest

Table 2 shows the correlation between variables. Among the socio-demographic and medical variables, number of children (*r* = −0.22, *p* < 0.01), age of the eldest children (*r* = −0.16, *p* < 0.05), and perceived health (*r* = −0.17, *p* < 0.01) had a negative correlation, while duration of the HIV status (*r* = 0.25, *p* < 0.01) had a positive correlation with participants’ intention to disclose their HIV status at 1-year follow-up. Furthermore, positive outcome expectations (*r* = 0.25, *p* < 0.01) had a positive correlation, while reward responsiveness (*r* = −0.17, *p* < 0.01) showed a negative correlation with intention to disclose at 1-year follow-up. These variables were entered in the hierarchical regression analysis.

**TABLE 2:** Correlation between variables (China, 2019–2022).

	1	2	3	4	5	6	7	8	9	10	11	12	13
1. Age	—												
2. Ethnicity	−0.03	—											
3. Education level	−0.04	0.10	—										
4. Marital status	0.16*	0.02	0.01	—									
5. Monthly household income	−0.11	0.10	0.26**	−0.17**	—								
6. Number of children	−0.17*	−0.12	−0.24**	−0.26**	−0.08	—							
7. Age of the eldest child	0.51**	0.08	−0.02	0.16*	−0.11	0.04	—						
8. Gender of the eldest child	0.01	−0.08	0.10	−0.04	0.10	0.10	−0.07	—					
9. Duration of HIV	0.16*	0.04	0.15*	0.03	−0.02	−0.18**	0.04	−0.02	—				
10. Perceived Health	−0.20**	0.01	0.08	−0.12	0.11	0.17**	−0.15*	−0.01	−0.21**	—			
11. Positive outcome expectations	0.02	0.05	0.06	0.08	0.01	−0.19**	0.02	0.02	0.03	−0.06	—		
12. Reward responsiveness	−0.17**	0.01	−0.04	−0.19**	0.13*	0.26**	−0.12*	−0.09	−0.10	−0.24**	0.08	—	
13. Intention to disclose HIV	0.06	−0.11	0.07	−0.01	−0.03	−0.22**	−0.16*	0.06	0.25**	−0.17**	0.25**	−0.17**	—

### Main Effect of Positive Outcome Expectations and Reward Responsiveness

Results from hierarchical regression analysis showed that in Block 1 where socio-baseline demographic and medical variables were entered, number of children (*β* = −0.17, *p* < 0.01), age of the eldest child, (*β* = −0.17, *p* < 0.01), and duration of HIV (*β* = 0.23, *p* < 0.01) significantly predicted intention to disclose HIV at follow-up. These variables accounted for 15% of total variance of intention to disclose HIV. In Block 2, where positive outcome expectations and reward responsiveness were added, positive outcome expectations had a positive association (*β* = 0.22 *p* < 0.01), while reward responsiveness had no significant association with intention to disclose HIV at follow-up after adjusting for socio-demographic variables and medical variables. These two additional variables accounted for 6% of total variance of intention to disclose HIV. In Block 3 where the interaction term of positive outcome expectation and reward responsiveness was added to the model, the interaction term was significant (*β* = 0.20 *p* < 0.01) and contributed to an additional 4% of total variance of intention to disclose HIV. The overall model explained 25% of variance of intention to disclose HIV at follow-up, F(7, 234) = 11.00, *p* < 0.001 (Table 3).

**TABLE 3:** Hierarchical regression analysis of BAS reward and positive outcome expectations on intention to disclose among women living with HIV (China, 2019–2022).

	Block 1			Block 2			Block 3		
	B	*β*	(95% CI)	B	*β*	(95% CI)	B	*β*	(95% CI)
Number of children	−0.20**	−0.17**	[−0.34, −0.06]	−0.11	−0.10	[−0.27, 0.03]	−0.11	−0.09	[−0.25, 0.04]
Age of the eldest child	−0.37**	−0.17**	[−0.63, −0.11]	−0.42**	−0.19**	[−0.65, −0.15]	−0.37**	−0.17**	[−0.62, −0.12]
Duration of HIV	0.36***	0.23***	[0.17, 0.56]	0.37***	0.22***	[0.21, 0.58]	0.35***	0.22***	[0.17, 0.54]
Perceived health	−0.24	−0.12	[−0.49, 0.01]	−0.20	−0.10	[−0.45, 0.05]	−0.21	−0.10	[−0.45, 0.03]
Positive outcome expectations (centered)				0.26***	0.22***	[0.12, 0.40]	0.26***	0.22***	[0.12, 0.39]
BAS reward (centered)				−0.13	−0.11	[-0.28, −0.01]	−0.07	−0.06	[−0.22, 0.07]
Positive outcome expectations (centered) x BAS reward (centered)							0.22**	0.20**	[0.09, 0.34]
DF	4			6			7		
F	10.66***			10.39***			11.00***		
Adjusted R square	0.15			0.21			0.25		

**p < 0.01, ***p< 0.001.

### Moderation Effect of Reward Responsiveness on the Association Between Positive Outcome Expectations and Intention to Disclose

**FIGURE 1:**
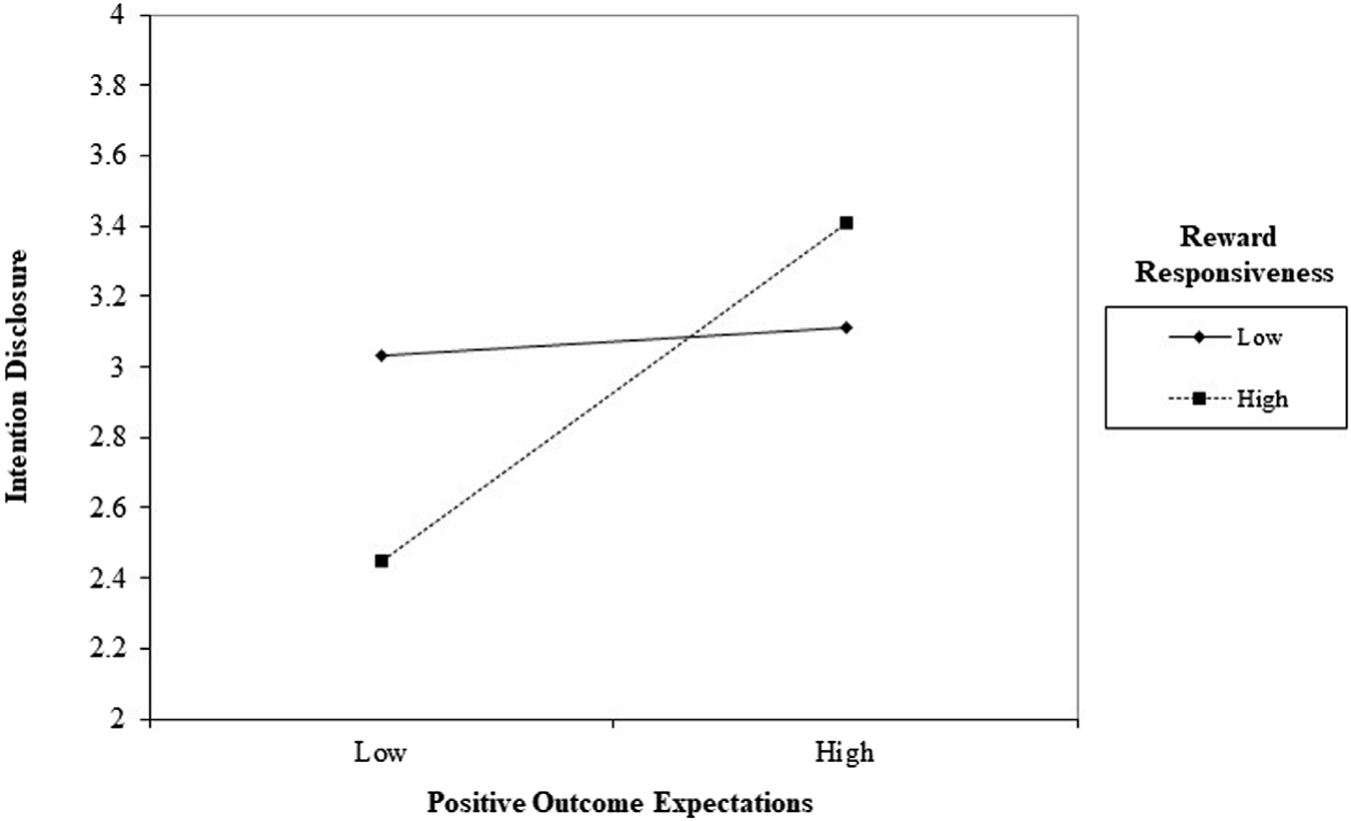
Interaction effect of positive outcome expectations and reward responsiveness on intention to disclose HIV among women living with HIV. (Note: 1 standard deviation below and 1 standard deviation above are used for low and high values respectively) (China, 2019-2022).

Results of the moderation analysis showed that reward responsiveness strengthened the positive relationship between positive outcome expectations and HIV disclosure intention. For participants with a low level of reward responsiveness, the level of positive outcome expectations had a small positive effect on their HIV disclosure intention. For participants with high reward responsiveness, their HIV disclosure intention significantly varied in accordance with the level of positive outcome expectations (Figure 1).

## Discussion

The current study examined the relationship between positive outcome expectations, reward responsiveness, and intention to disclose HIV to children among women living with HIV in China. The initial screening revealed that 113 out of 382 mothers living with HIV (29.5%) have disclosed their HIV status to at least the eldest child, which is comparable to the findings from previous studies (23%) [44]. For participants who have yet to make the HIV disclosure, age of the eldest child was negatively associated with the intention to disclose. In contrast, studies reported that children to whom HIV status was disclosed were often older [45, 46]. A possible explanation is that mothers with older children in this study have already concealed their HIV status for a long time, which demonstrated consistent reluctance to make the disclosure. Mothers with younger children have not yet encountered much stigma related to the issue, therefore held a more open attitude.

Duration of HIV status was positively associated with intention to disclose. This echoes with previous studies that mothers who have had their HIV diagnosis for a long time were more inclined to plan to disclose their HIV status to their children [45]. Results from this study found a negative relationship between duration of HIV status and one’s perceived health, which could serve as a practical reason for maternal HIV disclosure.

Results of our study showed that positive outcome expectations were positively associated with intention to disclose HIV for Chinese women with HIV. Findings are in line with studies that highlight the important role of positive outcome expectations on disclosure [23, 47]. Believing that HIV disclosure would result in positive outcomes can serve as an incentive for the behavior, therefore motivating individuals to disclose. HIV disclosure intention could possibly be increased by altering expectations with regards to the possible positive consequences of disclosure.

To our knowledge, the present study was the first attempt to investigate the moderating role of reward responsiveness in the relationship between positive outcome expectations and intention to disclose HIV. Findings showed that the relationship between positive outcome expectations and HIV status disclosure intention was stronger among women with higher levels of reward responsiveness. Findings are consistent with the regulatory focus theory that women with higher reward responsiveness are more likely to exhibit approach motivation for activities that provide a sense of pleasure, and are more responsive to positive outcomes. Therefore women living with HIV who have higher level of reward responsiveness tend to focus more on the possible benefits and positive consequences of HIV disclosure than the possible costs and negative consequences.

### Implications for Practice and Policy

Findings from the current study suggest ways to facilitate policies promoting intention to disclose HIV status to children. First of all, our study showed the predictive effect of positive outcome expectations on intention to disclose HIV status. In agreement with previous studies, campaigns educating public of the positive side of HIV disclosure would be useful to elevate peoples’ intention to disclose.

Furthermore, the current study highlights the importance of individual profiles when promoting intention to disclose HIV. While positive beliefs on the consequence of HIV disclosure generally improve the intention to disclosure, this might be particularly effective on individuals who are sensitive to positive emotions and reinforcement. Message framing should be considered to be compatible with one’s tendency of motivation system [48], in order to foster HIV intention to disclosure. The regulatory fit theory suggested that the match between message framing and one’s motivational orientation serves to sustain the manner of pursuing the goal, which eventually brought about behavioral change [38, 49]. Alternatively, expectations of the negative consequences of concealing their HIV status may urge other individuals who tend to avoid negative impacts to make the disclosure. This may be further investigated in future studies.

### Limitations

The study was subject to some limitations. Participants were recruited from 8 clinics, who might not be representative of the overall population of women living with HIV in China. Over 90% of participants perceive their own health as fair or above, they may therefore view the disclosure of HIV status as less imminent. Finally, as the disclosure behavior was very low (N = 4) at 1-year follow-up, only intention to disclose was used as the outcome and the 4 participants who had disclosed at follow-up were excluded from the analysis. Although intention has been regarded as one of the most proximal determinants of behaviors [25, 26], intention-behavior gap exists and intention does not necessarily translate into behavioral performance [50]. Results require cautious interpretation when applying to the context of HIV disclosure, as it only involved the measure of intention.

### Conclusion

The current study examined the association between positive outcome expectations, reward responsiveness and intention to disclose HIV among women living with HIV in China. Findings suggest that one’s behavioral motivation system magnifies the effect of outcome expectations on the specific intention to disclose HIV status. Findings support the relevance of positive outcome expectations and reward responsiveness regarding intention to disclosure, and the importance of considering the moderating role of reward responsiveness in HIV disclosure among women living with HIV in China.
